# The Potential Contribution of Dental Foci and Oral Mucositis to Febrile Neutropenia in Patients Treated With Myelosuppressive Chemotherapy for Solid Tumors and Lymphoma

**DOI:** 10.3389/froh.2022.940044

**Published:** 2022-06-30

**Authors:** Judith A. E. M. Zecha, Judith E. Raber-Durlacher, Alexa M. G. A. Laheij, Anneke M. Westermann, Jan de Lange, Ludi E. Smeele

**Affiliations:** ^1^Department of Oral and Maxillofacial Surgery, Amsterdam University Medical Centers, University of Amsterdam, Amsterdam, Netherlands; ^2^Department of Oral Medicine, Academic Center for Dentistry Amsterdam, University of Amsterdam and Vrije Universiteit, Amsterdam, Netherlands; ^3^Department of Preventive Dentistry, Academic Center for Dentistry Amsterdam, University of Amsterdam and Vrije Universiteit, Amsterdam, Netherlands; ^4^Department of Oncology, Amsterdam University Medical Centers, University of Amsterdam, Amsterdam, Netherlands; ^5^Academic Center for Dentistry (ACTA), Amsterdam, Netherlands; ^6^Department of Head and Neck Oncology and Surgery, Netherlands Cancer Institute- Antoni van Leeuwenhoek, Amsterdam, Netherlands

**Keywords:** febrile neutropenia, dental infection, oral mucositis, myelosuppressive chemotherapy, solid tumor

## Abstract

**Introduction:**

Febrile neutropenia (FN) is a potential life-threatening complication of myelosuppressive chemotherapy, particularly when induced by infection. There is evidence that FN can originate from the oral cavity, but its contribution to FN is largely understudied in patients treated for solid tumors. The aim of this study was to assess the prevalence of FN in these patients and to evaluate its relation with dental foci and oral mucositis.

**Material and Methods:**

A prospective longitudinal observational study was conducted. Patients diagnosed with solid tumors and lymphoma scheduled to be treated with myelosuppressive chemotherapy with an intermediate risk of developing FN were included. A pre-chemotherapy dental examination was performed and patients were followed during and after chemotherapy regimen. During subsequent hospital visits for chemotherapy administration, the oral cavity was inspected and oral mucositis (OM) was scored using the CTC-AE version 3.0. When patients presented with fever, a comprehensive full body examination including laboratory/microbiological/imaging investigation was performed.

**Results:**

Eighty-eight patients were included. Pre-chemotherapy, 39 patients (44.3%) were diagnosed with a dental focus. During chemotherapy, 46 patients developed OM (53.4%), of which 15 patients had a maximum score of grade II (ulcerative mucositis). Ten patients developed FN during the follow-up period. Patients with FN more often suffered from ulcerative OM compared to patients without FN; both FN and mucositis risk was associated with the myelotoxicity of chemotherapy. However, no relation could be established between the presence of dental foci prior to chemotherapy and the development of FN (*p* > 0.05).

**Conclusion:**

A significant relation was identified between ulcerative OM and FN, but no robust conclusions could be drawn with respect to a relationship between the presence of dental foci and FN.

## Introduction

Myelosuppressive chemotherapy (CT) is one of the modalities for treating solid tumors and lymphomas. Side effects include severe neutropenia during which patients are unable to mount a robust inflammatory response and are therefore at risk for infectious complications. Fever developing concurrently with neutropenia is classified as febrile neutropenia (FN) [1]. Depending on the degree of myelotoxicity of the CT regimen, patients have a low-, intermediate- or high risk of developing FN. Most CT regimens used for the treatment of solid tumors have an intermediate risk for FN development [1].

FN can prelude a life-threatening complication, particularly when caused by infection and should therefore be recognized at an early stage [2]. If a neutropenic patient presents with fever, a search for its cause should be performed consisting of history taking, physical examination and additional laboratory/microbiological/imaging investigation [3]. Non-infectious causes of FN include transfusion reactions, medication allergies and toxicities, vasculitis or other inflammatory conditions, and tumor(lysis)-related fever. Common sources of infection include the skin, urinary tract or lungs. However, as an infectious cause is documented clinically in only 20–30% of FN episodes and <30% of blood cultures is positive for microbial growth [4–11], the majority of fevers is classified as “fever of unknown origin”. Therefore, it is important to also consider other potential causes of FN, such as oral infection and inflammation.

The oral cavity contains teeth, periodontium, mucosa, and salivary glands, which may all act as foci of infection and inflammation and thus may induce FN. An oral focus is defined as a pathologic process in the oral cavity that does not cause major infectious problems in healthy individuals, but can lead to severe local or systemic infection under certain circumstances [12]. Pericoronitis, dental abscesses, infections associated with retained root tips, and apical periodontitis are potential dental foci. Apical periodontitis is a periradicular infection due to profound caries, with a reported prevalence of 52% in the general population [13]. Periodontal diseases are common chronic inflammatory diseases of the tissues supporting the teeth. Gingivitis is characterized by inflammation of the gingiva without loss of periodontal attachment, whereas periodontitis affects the deeper parts of the periodontium and is associated with alveolar bone loss [14]. Chronic gingivitis is seen in 40–50% of the population [15], whereas severe periodontitis is present in 7.0–10.8% of the population of Western countries [16]. These chronic infections may become acute and present with pain, redness and swelling, but during myelosuppression these signs and symptoms can be muted and may remain undiagnosed. Nevertheless, periodontal foci can be a cause of FN and infectious complications in myelosuppressed cancer patients [17, 18].

Furthermore, patients may have dental implants and develop peri-implant mucositis and peri-implantitis, which may have systemic implications similar to periodontal diseases [19].

A common side effect of CT and another possible oral cause of FN is oral mucositis (OM). OM is defined as mucosal inflammatory changes induced by cancer therapies, ranging from erythema to extensive ulcerations most often manifesting at the buccal and labial mucosa, ventral tongue, floor of mouth and soft palate [14]. Ulcerative OM may act as a portal of entry for oral microorganisms and inflammatory products into the bloodstream and may therefore contribute to FN (reviewed in 14).

A pre-transplant oral examination and elimination of oral foci is standard of care in high risk patients treated with myeloablative CT followed by stem cell transplantation or in patients diagnosed with head and neck cancer. However, in many cancer centers, pre-treatment screening for dental foci is not systematically performed in patients diagnosed with a solid tumor scheduled for CT regimens with an intermediate risk of developing (febrile) neutropenia. Since the cause of FN often remains unidentified and oral foci may be easily overlooked, oral examination before the start of CT may also be indicated in these patients as this may contribute to FN diagnosis and management. Yet, the evidence for a relationship between oral foci and FN in chemotherapy patients with intermediate FN risk is scarce. Therefore, the present study was aimed to assess the prevalence of FN and to evaluate its relation with dental foci and oral mucositis in patients treated with myelosuppressive chemotherapy for solid tumors or lymphoma.

## Materials and Methods

This study was performed according to the principles stated in the World Medical Association declaration of Helsinki 2018, at the Department of Oral and Maxillofacial Surgery and the Department of Oncology of the Amsterdam University Medical Center, location AMC. The Institutional Review Board approved this study (NL53440.018.15).

All participants received comprehensive information of the study aims and design and were informed when foci were diagnosed and advised to see their dentist after completion of CT when blood cell counts had normalized. All potential oral foci were noted on the medical records. This study had a prospective longitudinal observational design and took place between December 2015 and December 2020.

Patients ≥18 years, with a (partial) natural dentition and/or dental implants, no prior head and neck radiotherapy, diagnosed with a solid tumor (outside of the head and neck region) or lymphoma and scheduled for CT treatment with an intermediate risk of FN [1] were eligible for inclusion. Patient demographics including gender, age, body mass index (BMI), intoxications, American Society of Anesthesiologists (ASA) classification, World Health Organization (WHO) performance status and cancer diagnosis were retrieved from the medical files.

### Chemotherapy Regimens and Supportive Care Measures

Chemotherapy regimens were noted, including the number of planned CT cycles and supportive care measures (i.e., granulocyte colony stimulating factor, antibiotics, and anti-fungal/viral therapy). Adaptations to this treatment plan were also registered. Dose delay was defined as a delay of planned chemotherapy for more than 3 days; dose reduction was defined as one dose or more administered that was 85% or less of the initially planned dose [20]. A chemotherapy cancellation was defined as a initially planned dose that was not given at all.

Despite the strict inclusion criteria for FN risk [1], the actual risk of neutropenia varied. We therefore divided the group in relatively low- and relatively high risk of myelotoxicity (see Appendix 1). This classification was performed by an experienced oncologist (AW).

### Pre-chemotherapy Oral Screening

Prior to the start of CT an oral examination took place consisting of the following:

- Evaluation of dental mindedness (dental visits, oral hygiene habits) and oral complaints over the last 3 months
- Intra-oral screening for dental and/or mucosal pathology
- Periodontal screening using the Dutch Periodontal Screening Index [21].
- Screening for peri-implant mucositis and peri-implantitis
- Panoramic radiograph and selective peri-apical radiographs

All pre-existing dental and oral pathology that may contribute to the development of FN and infectious complications, was noted as an oral focus in accordance with the guidelines of the Dutch Association of Maxillofacial Surgery [22]. These included:

- Periodontal disease (DPSI 4; periodontal probing depth of >5 mm); (peri-implantitis was also considered as a focus)
- Profound dental caries
- Periapical pathology due to an infection of the root canal
- (Partially) impacted teeth
- Retained roots with surrounding pathology

Treatment of foci was only considered when symptomatic.

### Febrile Neutropenia

Febrile neutropenia was defined as: temperature > 38.5°C or two consecutive readings of >38.0°C for 2 h and an absolute neutrophil count < 500/μL or expected to fall below this threshold [3, 9]. When FN was diagnosed, laboratory- and/or radiological-results (including hematological full blood count, infection panel, urine sediment and chest X-ray) working diagnosis and treatment were noted. Blood cultures were checked for the presence of microbial growth after 2 days. Sepsis/septic shock and/or death was also noted.

### Assessment of Oral Mucositis and Other Oral Pathology During Chemotherapy Regimen

Oral mucositis was scored according to the CTC-AEv3.0 [23], during and after the planned CT regimen. All examiners were trained in reliable and consistent OM scoring and received an instruction card. The highest OM score during the observation period was used for analysis. When a patient presented with fever, OM was also assessed. Moreover, patients were examined for oral fungal and recrudescent herpes simplex virus infection and acute exacerbations of dental infection. The diagnosis was made on clinical findings, when deemed necessary microbiological investigations were performed.

### Statistical Analysis

The data were analyzed using the statistical package IBM SPSS for Windows (Version 26.0, IBM Corp, Armonk, NY, USA). Patients were divided into two groups; patients who developed FN and patients who did not develop FN. Differences between both groups and relations between the presence of FN, dental foci and OM were calculated using the Chi-Square test. A *p*-value < 0.05 was considered significantly different.

## Results

A total of 159 patients was eligible for inclusion. Of these, 93 patients agreed to participate and signed informed consent. Reasons for not participating included: the study was too burdensome (*n* = 9), logistical reasons (e.g., participating in other trials, already started with CT, not reachable) (*n* = 14), not eligible according to inclusion criteria (*n* = 5)„ dental anxiety (*n* = 5) or no reason recorded (*n* = 33).

**Figure 1:**
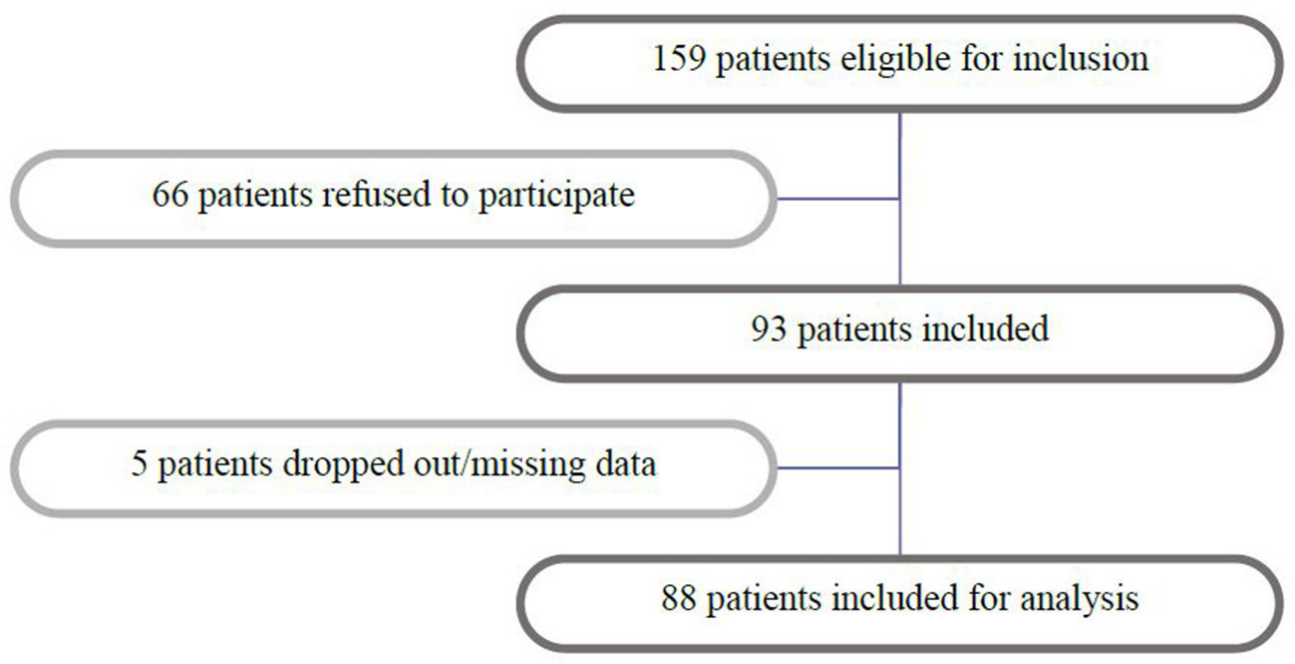
Patient inclusion.

In five patients OM scores were missing; one patient did not want to participate any longer, and four patients had progressive illness or no response to CT and started palliative care without any hospital visits. Finally, 88 patients were included for analysis (Figure 1).

Patient demographics are summarized in Table 1 and in Appendix 2 the patient group is divided in non FN and FN. Sixty-two patients (71%) were female and the mean age was 53.5 (±15.0) years. The majority of patients had a WHO performance status score of 0 prior to the start of CT. Gynecologic tumors were most frequently diagnosed (48%), followed by upper GI tract tumors (20.5%). The CT regimens were reviewed and divided in two subgroups based on myelotoxicity risk as described earlier (Appendix 1). Fifty-four patients had a CT regimen classified as having a relatively low risk for FN (61.4%), and 34 as having a relatively high FN risk (38.6%). A statistical difference was seen in CT-regimen and dose reduction between the FN-group and non FN-group.

**Table 1:** Patient demographics, tumor and treatment characteristics.

		**No. of patients**	**Percentage**
		**(*N*)**	**(%)**
**Patient demographics (*N* = 88)**			
Gender	Male	26	29.5
	Female	62	70.5
Age	Mean 53.5 years		
	Range 18–78 years		
	SD 15.0		
BMI	Mean 25.4		
	Range 16.8–44.3		
	SD 5.6		
Smoking	Yes	14	15.9
	No	53	60.2
	Quit	21	23.9
Alcohol use	Yes	29	33
	No	59	67
ASA classification	ASA I	50	56.8
	ASA II	33	37.5
	ASA III	5	5.7
WHO performance status	WHO 0	53	60.2
	WHO 1	32	36.4
	WHO 2	3	3.4
**Tumor and treatment characteristics (*N* = 88)**			
Tumor subgroup	Gynecological	42	47.7
	Upper GI tract	18	20.5
	Sarcoma	11	12.5
	Urinary tract	6	6.8
	Lymphoma	5	5.7
	Breast	4	4.5
	Lower GI tract	2	2.3
CT-regimen	Relatively high risk	34	38.6
	Relatively low risk	54	61.4
Treatment goal	Curative	59	67
	Palliative	29	33
Prophylactic G-CSF	Yes	16	18.2
	No	72	81.8
Dose reduction	Yes	19	21.6
	No	69	78.4
CT cycles alterations	Delay	20	22.7
	Cancellation	20	22.7
	No alterations	48	54.5

*BMI, Body Mass Index; ASA, American Society of Anesthesiologists; WHO, World Health Organization; GI, Gastro Intestinal; CT, chemotherapy; G-CSF, Granulocyte-colony stimulating factor*.

The mean follow up time was 94 days (range 15–200). Patients received on average five CT cycles (range 1–10). Twenty patients received less CT cycles than planned. Reasons included progressive illness, complications due to CT toxicity, surgical intervention and death.

### Oral Assessment Pre-chemotherapy

At the pre-chemotherapy oral screening, 64% of the patients reported visiting the dentist twice a year, 23.9% once a year and 12.5% visited the dentist never or sporadically. All patients but one, reported performing oral hygiene measures at least once daily. None of the patients reported having any acute oral complaints. See Table 2 for additional information about oral mindedness.

**Table 2:** Oral mindedness and dental visits.

		**No. of patients (*N*) & No. of smokers (*n*)**	**Percentage (%)**
**Oral mindedness (*N* = 88, *n* = 14)**			
Brushing	>Twice a day	6 (3)	6.8
	Twice a day	67 (7)	76.1
	Daily	14 (4)	15.9
	Unknown	1 (0)	1.1
Tooth picks	Daily	21 (2)	23.9
	Weekly	15 (2)	17
	Monthly	3 (0)	3.4
	Never	49 (10)	55.7
Flossing	Daily	7 (0)	8
	Weekly	10 (0)	11.4
	Monthly	1 (0)	1.1
	Never	70 (14)	79.5
Interdental brushes	Daily	24 (2)	27.3
	Weekly	10 (1)	11.4
	Monthly	1 (0)	1.1
	Never	53 (11)	60.2
Mouthwash	Daily	4 (0)	4.5
	Weekly	6 (1)	6.8
	Monthly	2 (0)	2.3
	Never	76 (13)	86.4
**Dental visits (*N* = 88, *n* = 14)**			
Dentist	Twice a year	56 (6)	63.6
	Once a year	21 (4)	23.9
	Sporadically	7 (2)	8
	Never	4 (2)	4.5
Oral hygienist	Twice a year or more	36 (4)	40.9
	Once a year	14 (2)	15.9
	Sporadically	6 (2)	6.8
	Never	29 (6)	33
	Unknown	3 (0)	3.4

Thirty-nine patients (44.3%) had a dental focus at the pre-chemotherapy evaluation. Dental foci were significantly more present in smokers (Chi-square, *p* = 0.03).

Of these, 25 had a periodontal focus, 25 had peri-apical pathology and one patient had peri-implantitis. Sixteen patients had more than one oral focus (Table 3). No dental interventions prior to the start of the CT were performed.

**Table 3:** Dental foci pre-chemotherapy, oral mucositis and oral mucosal infections during the course of chemotherapy (*N* = 88).

		**No. of patients**	**Percentage (%)**
**Dental foci prior to the start of chemotherapy**			
Yes		39	44.3
	Periodontal	25	28.4
	Peri-apical	25	28.4
	Profound caries	6	6.8
	Retained root tips	7	8
	Partial impacted teeth	2	2.3
	Peri-implantitis	1	1.1
	Multiple dental foci	16	18.2
No		49	55.7
Total		88	100
**Oral mucositis and oral mucosal infections during chemotherapy cycles**			
Oral Mucositis	Grade I	32	36.4
	Grade II	15	17
	No mucositis	41	46.6
Oral mucosal infections	Oral fungal infection	8	9.1
	Oral herpes simplex infection	1	1.1
	Other	20	22.7
	None	58	65.9

### Febrile Neutropenia

Sixteen patients developed febrile episodes during CT treatment, of which 10 met the criteria of FN diagnosis. Six patients developed fever but were not neutropenic; in two of these patients the fever was diagnosed as a reaction to medication, whereas three patients developed non-neutropenic fever induced by a clinically-documented non-oral infection (i.e., pneumonia, pleura empyema and influenza), and one had fever of unknown origin.

In patients who developed FN (*n* = 10), 11 FN episodes were further analyzed (Table 4). The median number of CT cycles before developing FN was two and FN occurred 9.8 days after the most recent cycle was given. The mean neutrophil count was 0.11 × 10^9^/L; the median temperature was 39.0°C. None of the patients developed sepsis/septic shock or died as a result of FN.

**Table 4:** Characteristics of patients (*n* = 10), in which 11 episodes of febrile neutropenia developed during chemotherapy cycles.

		**No. of patients**	**Percentage (%)**
Gender	Male	3	30
	Female	7	70
Age	Mean: 46.9 years (range 18–78)		
Tumor subgroup	Gynecological	1	10
	Upper GI tract	2	20
	Sarcoma	4	40
	Urinary tract	1	10
	Lymphoma	1	10
	Breast	1	10
	Lower GI tract	–	–
CT-regimen	Relative low myelosuppression risk	1	10
	Relative high myelosuppression risk	9	90
CT-cycles	Mean: 2 cycles (range 1-6)		
Temperature	Mean: 39.0°C (range 38.4–40.6°C)		
Neutrophil count	Mean: 0.11 × 109/L (range 0.00–0.33)		
Source of infection (per episode)	Urinary tract	2	18.2
	Airway	2	18.2
	Fever of unknown origin	7	63.6
Blood cultures (per episode)	Positive	1	9.1
	Negative	10	90.9
Treatment (per episode)	Intravenous antibiotics	10	90.9
	Oral antibiotics	1	9.1

*GI, gastrointestinal; CT, chemotherapy*.

In seven out of these 11 FN episodes no clinical non-oral infection could be identified. In case an infection was documented, it was either a urinary tract infection (18.2%) or an airway infection (18.2%). In all but one patient, the blood cultures were negative. In one patient *Staphylococcus epidermidis* was cultured. This patient had no pre-existent oral foci or OM at the time of the FN episode and a clinical infection was not identified. Nine of the 10 FN patients were treated with intravenous antibiotics, Augmentin + Ceftazidim being the first choice.

### Oral Mucositis, Dental Foci, and Febrile Neutropenia

During CT cycles, OM was recorded six times per patient on average (range 2–15). We followed a standardized protocol to score OM. Nevertheless, the length of the CT treatment varied among patients. This explains the wide range of number of OM evaluations. Overall, 47 patients developed OM (53.4%), of which 15 patients (17.0%) had a maximum score of grade II (Table 3). No grade III and IV OM were observed. In the majority of patients, OM occurred for the first time after administration of the first CT cycle (51.1%). Of patients receiving a relatively high risk CT regimen, 67.5% developed OM during any of the CT cycles, of which 22.4% developed ulcerative (grade II) OM.

Of the 10 patients who developed FN, four patients had OM during the FN episode of which one had grade II OM. In three patients the OM score was not noted during the FN episode. Patients with FN had a significantly higher chance of having more severe OM at any time during the course of their treatment (Chi-square, *p* = 0.005), a relatively higher myelotoxic CT regimen (Chi-square, *p* = 0.000), and more dose reductions (Chi-square, *p* = 0.02) compared to patients without FN. No significant relation was found between the development of FN and CT delays/cancellations (Chi-square, *p* > 0.05), Table 5.

**Table 5:** Dental foci, oral mucositis, febrile neutropenia and unplanned CT modifications (*N* = 88).

	**OM—Grade 0**	**OM—Grade I**	**OM—Grade II**	**Total**
FN	1	4	5	10
No FN	40	28	10	78
Total	41	32	15	88
**Chi-square: *p* = 0.005**				
	High risk CT-regimen	Low risk CT-regimen	Total	
FN	9	1	10	
No FN	25	53	78	
Total	34	54	88	
**Chi-square: *p* = 0.000**				
	Dose reduction	No dose reduction	Total	
FN	5	5	10	
No FN	14	64	78	
Total	19	69	88	
**Chi-square: p = 0.02**				
	Dental focus	No dental focus	Total	
FN	3	7	10	
No FN	36	42	78	
Total	39	49	88	
**Chi-square: *p* = 0.333**				
	CT delay	CT cancellation	No alterations	Total
FN	3	2	5	10
No FN	17	18	43	78
Total	20	20	48	88
**Chi-square: *p* = 0.843**				

*OM, oral mucositis; FN, febrile neutropenia; CT, chemotherapy*.

A dental focus was identified before the start of CT in three patients who presented with FN, however no significant relation could be identified between developing FN and the presence of a dental focus before CT (Chi-square, *p* > 0.05) (Table 5). Nevertheless, in one of these patients an asymptomatic partially impacted wisdom tooth became acutely painful during the FN episode. In another patient with FN without an evident non-oral cause, multiple periodontal and periapical foci were present prior to the start of the CT regimen. These oral sources of infection and inflammation may have induced fever.

Patients receiving CT-regimens classified with a relative high myelosuppression risk developed significantly more severe OM, compared to patients that underwent low-risk CT (Chi-square, *p* = 0.001). No significant relation was found between OM and dose reductions, delay or cancellation of CT (Chi-square, *p* = 0.580, *p* = 0.449). No significant relation was identified between the presence of an oral focus before CT and the development of OM during CT (Chi-square, *p* = 0.714), see Table 6.

**Table 6:** Oral mucositis, dental foci and unplanned CT modifications (*N* = 88).

		**OM—Grade 0**	**OM—Grade I**	**OM—Grade II**	**Total**
CT-regimen	High risk	8	15	11	34
	Low risk	33	17	4	54
	Total	41	32	15	88
**Chi-square: *p* = 0.001**					
Dose reduction	Yes	10	5	4	19
	No	31	27	11	69
	Total	41	32	15	88
**Chi-square: *p* = 0.580**					
CT cycle alterations	Delay	7	8	5	20
	Cancellation	11	8	1	20
	No alterations	23	16	9	48
	Total	41	32	15	88
**Chi-square: *p* = 0.449**					
Dental focus	Yes	18	13	8	39
	No	23	19	7	49
	Total	41	32	15	88
**Chi-square: *p* = 0.714**					

*OM, oral mucositis; CT, chemotherapy*.

## Discussion

The aim of the study was to assess the prevalence of FN in patients treated with myelosuppressive chemotherapy for a solid tumor or lymphoma and to evaluate its relation with dental foci and oral mucositis. Prior to the start of CT, 44.1% of patients had one or more dental foci. During CT, ten patients (11.4%) developed FN, and 15 patients (17.0%) developed ulcerative OM.

A statistically significant relation was found between the presence and severity of OM and developing FN, suggesting that patients with ulcerative OM are also at risk for developing FN. Patients treated with CT regimens with a relatively high risk of myelosuppression had a significant higher risk of developing OM compared to those treated with relatively low risk CT regimens. We found no statistically significant relation between the presence of dental foci before the start of CT and the development of FN or OM during CT.

In this study, a total of 53.4% of patients developed OM (grade I and II), which is higher than reported in the literature, in which an incidence of 15.0–42.3% (all mucositis grades) was reported in patients treated for solid tumors and lymphoma [24–29]. Nevertheless, Jones et al. [26] reported an OM rate of 60% in patients receiving TAC (Taxotere-Adriamycin-Cyclophosphamide) chemotherapy for breast cancer. Raber-Durlacher et al. [30] found an incidence of OM in 31%, of which 16.7% had OM grade II in a retrospective study in patients treated with CT for solid tumors. Whereas the overall incidence in the present prospective study was higher, the incidence of OM grade II is in accordance with our results. OM grade II is likely less underscored as it is painful and characterized by ulcerations, facilitating its identification [28].

A relationship between the incidence and severity of OM and the development of fever has been reported in several studies [11, 28, 29], similar to our findings. van der Velden et al. [11] introduced the term “febrile mucositis” based on their observations in stem cell transplantation recipients; the mucosal barrier may be damaged due to CT leading to the generation of inflammatory cytokines (IL-1 and IL-6) and a disturbed host-microbe interaction may arise, which may lead to fever. Our study provides additional support for a link between OM and FN in patients with solid tumors. It should be noted, however that mucosal injury may occur throughout the whole gastrointestinal tract and our study was only directed to oral mucosal injury.

Among other risk factors, the prevalence of OM is related to the CT regimen administered [14, 28, 31]. Kishimoto et al. [31] found a significant higher rate of OM in patients receiving CT regimens causing more severe myelosuppression. Although a direct relationship between peripheral neutrophil numbers and OM risk was not established, their study confirmed that the higher the myelotoxicity of CT regimens, the higher the risk to develop OM. Our study population falls within an intermediate risk of myelosuppression [1], but there was also differentiation possible in this group based on myelosuppression risk. Thus, in order to estimate OM risk, it is advisable to look more closely at the myelotoxicity of the intended CT regimen.

Even though most of the patients visited the dentist on a regular base, a high percentage (44.3%) of patients had an oral focus prior to the start of the chemotherapy, although lower than reported in the literature [13, 15, 32]. In most cases, asymptomatic chronic dental foci seem not to cause infectious problems in healthy individuals [12]. However, patients who become myelosuppressed may be at risk for exacerbation of an asymptomatic infection, which may lead to local or systemic inflammation and infection [12]. In our study, one patient with FN developed an acute exacerbation of a dental focus, while in another patient with FN without an evident non-oral cause, multiple periodontal and periapical foci were present. Nevertheless, we found no statistically significant relation between the presence of dental foci and FN during CT, which may be explained by the relatively small number of included patients.

In patients receiving intensive CT for hematologic malignancies, Spijkervet et al. [12] and Schuurhuis et al. [33] proposed to only eliminate oral foci with acute signs/symptoms or chronic infections with an exacerbation during the previous 3 months. In contrast, Kishimoto et al. [31] reported a significantly higher incidence of odontogenic infections during CT regimens in patients treated with CT for hematologic malignancies who did not complete their dental treatment prior to the start of CT, however this incidence was not related to the grade of myelosuppression. This suggests that odontogenic infections can occur in any kind of myelosuppressive CT. Raber-Durlacher et al. [17] suggested that both chronic and exacerbating periodontal diseases may induce fever and infectious complications in patients receiving intensive high-dose CT regimens, but clinical studies aimed to identify such relationships are difficult to perform.

Nevertheless, more large prospective studies are necessary to allow any definitive conclusions about management recommendations on the treatment of oral foci in patients scheduled for myelosuppressive CT.

Our study had several limitations as we evaluated a relatively small number of patients and did not include age, comorbitities and performance status [1] in our analyses. Another limitation of the study is the low incidence of positive blood cultures in the patients who developed FN. Therefore, assessment of the potential contribution of the oral flora to bacteremia was not possibible.

Our group [14] conducted a review about the impact of the oral cavity in febrile neutropenia and infectious complications in patients treated with myelosuppressive CT. This may serve as a guidance in the management and prevention of oral complications in these patients. However, as concluded in this review, limited evidence is present about the implications of oral foci in patients treated with myelosuppressive CT for solid tumors and lymphoma. The present study may serve as a first step for further research in this area.

Although the number of included patients was not sufficient to draw robust conclusions, we identified a significant relation between the presence and severity of ulcerative OM and developing FN, suggesting that patients with ulcerative OM are also at risk for developing FN. Furthermore, we did not find a statistically significant relation between the presence of dental foci and FN during CT, pointing to the notion that chronic dental foci may not have to be aggressively eliminated before the initiation of CT in patients with solid tumors. Nevertheless, it is advisable to encourage patients to maintain good oral hygiene during CT, particularly when they will receive CT with a relatively high risk of myelosuppression. In order to draw robust conclusions about the potential role of dental foci and oral mucositis in the development of FN, larger prospective studies are needed.

## Data Availability Statement

The raw data supporting the conclusions of this article will be made available by the authors, without undue reservation.

## Ethics Statement

The studies involving human participants were reviewed and approved by Institutional Review Board, Amsterdam University Medical Centers. The patients/participants provided their written informed consent to participate in this study.

## Author Contributions

JZ, JR-D, and AL contributed to conception and design of the study. JZ organized the database and wrote the first draft of the manuscript. JZ and AL performed the statistical analysis. JR-D and AL wrote sections of the manuscript. All authors contributed to manuscript revision, read, and approved the submitted version.

## Conflict of Interest

The authors declare that the research was conducted in the absence of any commercial or financial relationships that could be construed as a potential conflict of interest.

## Publisher's Note

All claims expressed in this article are solely those of the authors and do not necessarily represent those of their affiliated organizations, or those of the publisher, the editors and the reviewers. Any product that may be evaluated in this article, or claim that may be made by its manufacturer, is not guaranteed or endorsed by the publisher.
