# Supplementary material for: The Potential Contribution of Dental Foci and Oral Mucositis to Febrile Neutropenia in Patients Treated With Myelosuppressive Chemotherapy for Solid Tumors and Lymphoma
Source: Front Oral Health. 2022 Jun 30;3:940044. doi: 10.3389/froh.2022.940044 (PMC9280026; doi:10.3389/froh.2022.940044)
Supplement: Supplementary file 1 [file Data_Sheet_1.PDF]

Appendix 1. Classification of chemotherapy regimen based on myelotoxicity

High Risk	Low Risk
5FU 400mg/m2 + continuously 2400mg/m2 for 46h, Oxaliplatin 85mg/m2, Irinotecan 180mg/m2, Leucovorin 400mg/m2	Bevacizumab 10mg/kg, Doxorubicin 40mg/m2
Bleomycin 30USP, Cisplatin 20mg/m2, Etoposide 100mg/m2	Bevacizumab 15mg/kg, Carboplatin AUC 4, Gemcitabin 1000mg/m2
Bleomycin 10 USP, Dacarbazin 375mg/m2, Doxorubicin 25mg/m2, Vinblastin 6mg/m2	Bevacizumab 15mg/kg, Cisplatin 50mg/m2, Paclitaxel 175mg/m2
Capecitabin 1000mg/m2, Epirubicin 50mg/m2, Oxaliplatin 130mg/m2	Capecitabin 1000mg/m2, Oxaliplatin 65mg/m2, NAB-Paclitaxel 80mg/m2
Cisplatin 20mg/m2, Etoposide 100mg/m2	Capecitabin 1000 mg/m2, Oxaliplatin 130mg/m2
Cisplatin 60mg/m2, Doxorubicin 37,5mg/m2, MTX 12g/m2	Capecitabin 750mg/m2, Oxaliplatin 130mg/m2
Cisplatin 80mg/m2, Etoposide 100mg/m2	Carbazitaxel 25mg/m2, Prednison 5mg 2dd
Cisplatin 80mg/m2, Dactinomycin 2mg, Doxorubicin 30mg/m2	Carboplatin AUC 2, Paclitaxel 50mg/m2
Cyclophosphamide 300mg/m2, Prednison 40mg/m2, MTX intrathecal, Vincristin 2mg	Carboplatin AUC 4, Gemcitabin 1000mg/m2
Cyclophosphamide 600mg/2, Docetaxel 75 mg/m2	Carboplatin AUC5, liposomaal doxorubicin 30mg/m2
Cyclophosphamide 600mg/m2, Doxorubicin 60mg/m2, Paclitaxel 80mg/m2	Carboplatin AUC 6, Paclitaxel 175 mg/m2
Cyclophosphamide 600mg/m2, Dactinomycin 0,5mg, Etoposide 100mg/m2, MTX 300mg/m2, Vincristin 0,8mg/m2	Carboplatin AUC2, Paclitaxel 50mg/m2
Cyclophosphamide 750mg/m2, Doxorubicin 50mg/m2, MTX high dose, Rituximab 375mg/m2, Vincristin 2mg	Carboplatin + Paclitaxel + Trastuzumab + Pertuzumab
Cyclophosphamide 750mg/m2, Doxorubicin 50mg/m2, Prednison, Rituximab 375mg/m2, Vincristine 2mg	Cisplatin 40mg/m2
Dactinomycin 2mg, Ifosfamide 3gr/m2, Vincristine 2mg	Cyclophosphamide 60mg/m2, Doxorubicin 60mg/m2, Paclitaxel 80mg/m2
Docetaxel 75mg/m2, Gemcitabin 900mg/m2	Dacarbazin 375mg/m2, Doxorubicin 25mg/m2, Vinblastin 6mg/m2
Doxorubicin 37,5mg/m2, Ifosfamide 3000 mg/m2	Doxorubicin 75mg/m2
Doxorubicin 20mg/m2, Etoposide 150mg/m2, Ifosfamide 3000mg/m2, Vincristin 2mg	Doxorubicin 75mg/m2, Olaratumab 15mg/kg
Etoposide 100mg/m2, Ifosfamide 3000mg/m2, Vincristin 2mg	Gemcitabin 1000mg/m2, NAB-Paclitaxel 125mg/m2
Folfiri: 5FU + Irinotecan:Irinotecan 180mg/m2, Folinezuur 400mg/m2, Fluorouracil 400mg/m2	Liposomal Doxorubicin 45mg/m2
DA-EPOCH-R (Cyclophosphamide Etoposide, Prednisolon, Vincristin, Hydroxoanurubicine, Rituximab) + MTX it	

(5FU = 5-Fluoruracil, USP = United States Pharmacopeia, MTX = methotrexate, AUC = area under the curve, NAB = nanoparticle albumin-bound, It = intrathecal)
