# Supplementary material for: The Potential Contribution of Dental Foci and Oral Mucositis to Febrile Neutropenia in Patients Treated With Myelosuppressive Chemotherapy for Solid Tumors and Lymphoma
Source: Front Oral Health. 2022 Jun 30;3:940044. doi: 10.3389/froh.2022.940044 (PMC9280026; doi:10.3389/froh.2022.940044)
Supplement: Supplementary file 2 [file Data_Sheet_2.PDF]

Patient demographics (N=88)					
No Febrile neutropenia				Febrile neutropenia	
		No. of patients (N)	Percentage (%)	No. of patients (N)	Percentage (%)
Gender	Male	23	29.5	3	30.0
	Female	55	70.5	7	70.0
					Chi square p= 0.973
Age	Mean 54.0 years<br>Range 19-76 years<br>SD 14.3			Mean 49.3 years<br>Range 18-78 years<br>SD 19.8	
					Chi square p= 0.558
BMI	Mean 25.5<br>Range 16.8-44.3<br>SD 5.7			Mean 24.6<br>Range 18.3-30.8<br>SD 4.5	
					Chi square p= 0.332
Smoking	Yes	14	17.9	0	0.0
	No	48	61.5	5	50.0
	Quit	16	20.5	5	50.0
					Chi square p= 0.074
Alcohol use	Yes	24	30.8	5	50.0
	No	54	69.2	5	50.0
					Chi square p= 0.223
ASA classification	ASA I	44	56.4	6	60.0
	ASA II	30	38.5	3	30.0
	ASA III	4	5.1	1	10.0
					Chi square p= 0.756
WHO performance status	WHO 0	47	60.3	6	60.0
	WHO 1	29	37.2	3	30.0
	WHO 2	2	2.6	1	10.0
					Chi square p= 0.458

Tumor and treatment characteristics (N=88)					
No Febrile neutropenia				Febrile neutropenia	
		No. of patients (N)	Percentage (%)	No. of patients (N)	Percentage (%)
Tumor subgroup	Gynecological	41	52.6	1	10.0
	Upper GI tract	16	20.5	2	20.0
	Sarcoma	7	9.0	4	40.0
	Urinary tract	5	6.4	1	10.0
	Lymphoma	4	5.1	1	10.0
	Breast	3	3.8	1	10.0
	Lower GI tract	2	2.6	0	0.0
					Chi square p= 0.068
CT-regimen	Relatively high risk	25	32.1	9	90.0
	Relatively low risk	53	67.9	1	10.0
					Chi square p= 0.00
Treatment goal	Curative	50	64.1	9	90.0
	Palliative	28	35.9	1	10.0
					Chi square p= 0.101
Prophylactic G-CSF	Yes	12	84.6	4	40.0
	No	66	15.4	6	60.0
					Chi square p= 0.057
Dose reduction	Yes	14	17.9	5	50.0
	No	64	82.1	5	50.0
					Chi square p= 0.020
CT cycles alterations	Delay	17	21.8	3	30.0
	Cancellation	18	23.1	2	20.0
	No alterations	43	55.1	5	50.0
					Chi square p= 0.843
